# Motives to Have Sex: Measurement and Correlates With Sociodemographic, Sexual Life, and Psychosexual Characteristics

**DOI:** 10.3389/fpsyg.2021.645493

**Published:** 2021-07-12

**Authors:** Juan Ramón Barrada, Ángel Castro, Elena Fernández-del-Río, Pedro J. Ramos-Villagrasa

**Affiliations:** ^1^Department of Psychology and Sociology, Universidad de Zaragoza, Teruel, Spain; ^2^Department of Psychology and Sociology, Universidad de Zaragoza, Zaragoza, Spain

**Keywords:** sexual motives, university students, validation, Sexual Motivations Scale, sexual behavior

## Abstract

Knowledge of diverse sexual motivations can have profound implications for our comprehension of the causes, correlations, and consequences of sexual behavior. This study had two objectives: on the one hand, to determine the different motives why young Spanish university students have sex and their relationship with different sociodemographic and psychosexual variables and sexual behavior; on the other hand, to review and improve the psychometric properties of the Sexual Motivations Scale and validate it in Spanish. Participants were 805 university students of both sexes (78% women, 74% heterosexuals), aged between 18 and 26 years (*M*_*age*_ = 20.88), who completed a battery of online questionnaires. Significant associations were found between young people's sexual motives, especially the motives of coping, peer pressure, and enhancement, the sociodemographic variables (sex, age sexual orientation, relational status), sexual behavior (age of initiation), and psychosexual variables (sociosexuality, self-esteem as a sexual partner, satisfaction with sex life). Also, a new structure of the Sexual Motivations Scale was proposed, with the elimination of the factor of Self-Affirmation. The discussion highlights the relevance of the results obtained due to their implications in the promotion of sexual health, in addition to achieving the first instrument validated in Spanish for the evaluation of sexual motivations.

## Introduction

Until a few decades ago, the main reason admitted for having sex was reproduction (Meston and Buss, 2007). The first scientific literature on the subject extended the reasons for having sex, and other motives—such as pure pleasure, expressing emotional intimacy, or releasing stress—were considered (Leigh, 1989; Hill and Preston, 1996).

Motives for sex can predict different behavior patterns and some of those patterns can have negative consequences for health and well-being (Cooper et al., 1998). Therefore, the study and knowledge of people's sexual motivations are of interest to public health and health promotion (Hensel et al., 2017).

### Assessing Motivations for Sex

Traditionally, three perspectives have been used for the development of sexual motive questionnaires. First, as Hill and Preston (1996) noted 25 years ago, “many instruments have apparently been developed *ad hoc* for each study, with little attention to the reliability and validity of the measures” (p. 27). Second, some authors have used lists elaborated by their participants and have examined the psychometric properties of those questionnaires, as in the YSEX? scale (Meston and Buss, 2007). However, the factor structure of the YSEX? is unclear, at least in women with same-sex attraction (Armstrong and Reissing, 2014). Recently, a short form of this scale has been presented (Meston et al., 2020) although some dimensions showed a rather low reliability (minimum Cronbach's α = 0.42), and the model fit of factor analysis was also below the commonly used cut-off points (e.g., TLI = 0.86).

Third, some proposals are grounded on a more solid basis, such as, for example, classic personality, and social psychology theories (e.g., Leigh, 1989; Hill and Preston, 1996; Gravel et al., 2016). Leigh (1989) indicated the appropriateness of evaluating both the reasons for having and for not having sex. Hill and Preston (1996) pointed to the need to distinguish the type of incentives desired (those related to pleasure and reproduction and other socially-oriented ones such as affection and power) and whether individuals perceive themselves as the agent or the recipient of certain actions. The questionnaire developed by Gravel et al. (2016), based on the self-determination theory (Ryan and Deci, 2018), assesses intrinsic motivation, extrinsic motivation, and amotivation.

Considering all these proposals, from our point of view, the questionnaire with the most solid theoretical basis was that proposed by Cooper et al. (1998). Following a functional perspective in behavior and the philosophy of other classifications that serve to explain why people eat, smoke, or drink alcohol (Cooper et al., 2016), these authors proposed a taxonomy of motivations that arises from the response to two relevant axes. The first axis refers to whether the behavior is motivated by the desire to pursue positive-pleasurable consequences or to avoid negative-harmful consequences. The second axis considers whether the behavior is oriented toward an individual or a social objective.

Thus, crossing the indicated dimensions, four categories of sexual motives are created: (1) appetitive self-focused motivations, such as having sex to obtain physical or emotional pleasure (enhancement motives); (2) aversive self-focused motives, such as having sex to avoid or minimize negative emotions (coping motives); (3) appetitive social motives, such as having sex to reinforce intimacy with others (intimacy motives); and (4) aversive social motives, such as having sex to avoid isolation and/or to gain the approval of others (approval motives).

This classification inspired the first and, to date, one of the most commonly used instruments to assess sexual motivations: The Sexual Motivations Scale (SMS; Cooper et al., 1998). It is a self-administered questionnaire, targeting sexually active people, consisting of 29 items that evaluate how often they have sex for different reasons. In principle, the authors wanted to find evidence for the above-mentioned four-factor model, two appetitive and two aversive factors. However, after factor analysis, they found a better fit for a six-factor model, the four initially proposed factors plus two additional aversive ones. At the individual level, in addition to the coping motives, they proposed a factor of self-affirmation. While coping is the customary term used to specifically designate the regulation of negative affect, self-affirmation is intended to denote an increase of positive affect and self-worth. At the social level, they distinguished peer pressure and partner approval. Thus, the six factors proposed by Cooper et al. (1998) were: (1) Intimacy, (2) Enhancement, (3) Self-Affirmation, (4) Coping, (5) Peer Pressure, and (6) Partner Approval. All factors are composed of five items, except the last, which has four, and are rated on a Likert scale of 1 = *almost never/never* to 5 = *almost always/always*.

In its original development, the SMS showed good internal consistency, adequate convergent, divergent, and criterion validity, and adequate invariance across race, sex, and age (Cooper et al., 1998). Its structure and reliability were endorsed by the only validation of the instrument that could be located, also carried out in the United States, which included sexual minorities and cultural groups that had not been previously evaluated (Jardin et al., 2017). Jardin et al. (2017) tested measurement invariance concerning sex, age, race/ethnicity, sexual minority status, and relationship status.

The relevance of this instrument is reflected in its basis for many subsequent studies (cf. Patrick et al., 2011; Kenney et al., 2014; Snapp et al., 2014), and it has inspired the development of other scales in the field of sexuality. For example, Kenney et al. (2014) used it as a basis for evaluating a hookup motives questionnaire, whereas Patrick et al. (2011) used three of its subscales (Enhancement, Intimacy, Coping) and added other subscales to assess motives for and against sexual behavior.

### Correlates of Motivations for Sex

Within the correlations of the motives for having sex, the most repeated analysis is that of the differences between men and women. However, the results obtained are inconclusive. It has traditionally been considered that men grant more relevance to the pursuit of pleasure and physical gratification, whereas women attach greater importance to the motives of intimacy and emotional closeness (Carroll et al., 1985; Leigh, 1989; Cooper et al., 1998; Ott et al., 2006; Meston and Buss, 2007; Armstrong and Reissing, 2015; Wyverkens et al., 2018). However, women's motivations seem to have changed in recent years, more resembling those of men, with the pursuit of pleasure as the main objective (Ozer et al., 2003; Patrick et al., 2007, 2011).

Age, sexual orientation, and relationship status are also sociodemographic variables that have been linked to sexual motives. In terms of age, an evolution has been documented throughout youth, following the formation of identity (Cooper et al., 1998). This evolution shows that, as people age, the reasons related to intimacy become more important, more so than the reasons for self-affirmation and peer pressure, more characteristic of early adolescence (Ott et al., 2006). In the case of sexual orientation, despite being a relevant variable, research is scarce. Some motivations (e.g., procreation) make no sense in non-heterosexual relationships. Leigh (1989) concluded that heterosexuals attached more importance to reproduction, emotional closeness, and the partner's pleasure, whereas members of sexual minorities considered that the release of sexual tension was more important.

Relational status is a relevant variable for understanding the relationship between the reasons for having sex and sexual behavior (Cooper et al., 1998). The literature has shown that people who are more motivated by intimacy are more predisposed to seek and maintain committed relationships, whereas people who are motivated by other reasons can satisfy their needs just as well or better through casual sex (Armstrong and Reissing, 2015; Jardin et al., 2017). Gravel et al. (2016) found that self-affirmation was less relevant as a sexual motivation for those in a committed relationship.

The associations between motivations for sex and sexual behavior, especially in terms of risky practices, seem well-established (Cooper et al., 1998; Grossbard et al., 2007; Patrick et al., 2007). It has been found that individuals who have sex for enhancement perform more risky behaviors (e.g., earlier initiation, more sexual partners). Coping and peer-pressure motives are associated with more sexual partners, whereas self-affirmation and partner-approval motives are usually associated with a later initiation, fewer experiences, and fewer sexual partners (Aspden et al., 2010; Barber and Cooper, 2014; Jardin et al., 2017).

There are other psychosexual variables whose association with sexual motivations may be relevant, although the literature that addresses them is scarce. This is the case of sociosexuality, understood as the orientation toward sexual relations without commitment (Penke and Asendorpf, 2008). Sociosexuality, conceptualized as a tridimensional construct but measured as unidimensional until recent times (Barrada et al., 2018), has been positively related to all the motivations for having sex (Meston and Buss, 2007), except those of intimacy (Meston et al., 2020). The most powerful associations have been found with the enhancement motives, due to their relationship with casual sex and the pursuit of pleasure (Cooper et al., 1998).

Even more scarce is the literature on the relationship between motives and psychosexual well-being. The findings point to a possible relationship between enhancement and intimacy motives, sexual self-esteem (Townsend et al., 2019), and satisfaction with sex life (Impett and Tolman, 2006; Stephenson et al., 2011), but there are few studies.

### The Present Study

Due to the relevance of understanding young people's sexual motivations, this study aims to overcome some limitations in the literature. These limitations focus on the tools available to measure sexual motivations and their correlates, considering the cultural context of previous studies. Thus, firstly, as no similar study has been found outside of North America, we wished to examine similarities and differences in the psychometric properties of the SMS (Cooper et al., 1998), as well as the patterns of correlates of specific motives with demographics and psychosocial and sexual variables in a new culture and language group. Therefore, we wanted to examine a Spanish version of the SMS that can be used in future research.

Secondly, we wanted to analyze the internal structure of the SMS with a more robust and updated psychometric approach. The latest analysis (Jardin et al., 2017) used confirmatory factor analysis. This approach, where secondary loadings are fixed to zero, has been shown to potentially distort the recovered model when relevant cross-loadings are present (Asparouhov and Muthén, 2009; Sánchez-Carracedo et al., 2012; Marsh et al., 2014). These problems may not be reflected in the overall model fit. In line with this, we used structural equation models, which can better estimate the effect sizes, as measurement error is taken into account (Cole and Preacher, 2014).

Thirdly, we wanted to advance knowledge about motives for having sex among Spanish university students. University years represent a period of increasing freedom and autonomy for many students. These years are characterized by new experiences and relationships, some of which can prove to be risky for youth's health (Cooper, 2002; Eisenberg et al., 2012; Castro and Santos-Iglesias, 2016). Specifically, our goals were: (1) to determine the structure of their sexual motives; (2) to determine whether their motives for having sex differ as a function of sociodemographic variables (i.e., gender, age, sexual orientation, relational status); and (3) to investigate the relationship between sex motives and sexual behavior (i.e., frequency of masturbation, age of initiation), and other psychosexual variables—whose associations with motives have received very little attention in previous literature—, such as sociosexuality (understood and assessed as three-dimensional, formed by behavior, attitudes, and desire) and well-being (i.e., self-esteem as a sexual partner, satisfaction with sex life, preoccupation with sex). Importantly, to date, the different studies that have examined these associations have not examined all these variables simultaneously, so the comparability of effects was reduced, as each effect was derived from different samples.

## Method

### Participants and Procedure

The initial sample comprised 1,373 participants. Five inclusion criteria were used:

- Studying a university degree (81 participants excluded). We excluded these participants because they did not belong to our intended population.
- Aged between 18 and 26 years, based on criteria from previous studies with university samples [e.g., Barrada et al., 2019b, 2021; Barrada and Castro, 2020; Castro et al., 2020); 121 participants excluded]. We decided to maintain consistency across studies to reduce researchers' degrees of freedom and, thus, avoid potential *p*-hacking (Wicherts et al., 2016).
- Labeling themselves as woman or man (seven participants excluded). We excluded these participants because the very small sample size prevented us from any analysis of this group.
- Having had any sexual intercourse with penetration in their lifetime (217 participants excluded), as the wording of the items of the SMS assumed sexual experience. Cooper et al. (1998) included in their study “sexually experienced” participants, although no information was provided about what could be considered as “experienced.” Jardin et al. (2017) provided no information about inclusion/exclusion criteria in their study. By limiting our attention to those with penetrative sex, we were narrowing the conception of sexual life, as penetration is not the only way to have a sexual experience, but we were establishing a clearer operationalization of what could be considered as “sexually experienced.” Otherwise, what constitutes a sexual experience is not homogeneously considered by all participants (Carpenter, 2001; Bersamin et al., 2007; Hans et al., 2010).
- Correctly answering a control question (142 participants excluded; see below).

After applying these criteria, the final sample comprised 805 Spanish university students (78% women, 22% men), aged between 18 and 26 (*M* = 20.88, *SD* = 2.15). Of these participants, 74.0% described themselves as heterosexual, 20.5% as bisexual, 4.1% as homosexual, and 1.4% as other orientations. Due to the small sample sizes of non-heterosexual participants, those participants were grouped into a sexual minority category (26.0%). Of the participants, 59.5% had a partner at the time of the study.

Data were collected through the Internet with Google Forms in November 2018. The link to the survey was circulated through the e-mail distribution lists of the students of the authors' university. The survey remained open for 14 days. Participants provided informed consent after reading the description of the study, where the anonymity of the responses was clearly stated. This procedure was approved by the Ethics Review Board for Clinical Research of the region (PI18/058). This sample has been used in previous research (Fernández del Río et al., 2019), although other variables and research questions were considered there.

### Instruments

#### Sociodemographic and Sexual Behavior Questionnaire

We asked participants about their gender (woman, men, other), age, sexual orientation (heterosexual, homosexual, bisexual, other), if they were currently in a relationship, frequency of masturbation (times per day), and age of sexual initiation.

#### Sexual Motivations Scale

Only the participants who had had any sexual intercourse with penetration in their lifetime completed the Sexual Motivations Scale (SMS; Cooper et al., 1998). This instrument has 29 items that assess motives for having sex based on six dimensions: Intimacy (e.g., “To become closer to your partner”; Cronbach's α = 0.90 –all reported alphas correspond to values obtained with the current sample), Enhancement (e.g., “Just for the excitement of it”; α = 0.78), Self-Affirmation (e.g., “Because it makes you feel more self-confident”; α = 0.76), Coping (e.g., “To help you deal with disappointment in your life”; α = 0.81), Peer Pressure (e.g., “Because you worry that people will talk about you if you don't have sex”; α = 0.88), Partner Approval (e.g., “Out of fear that your partner won't love you any more if you don't”; α = 0.87). Participants rated how often they had sex for each reason on a five-point Likert-type scale ranging from 1 = *almost never/never* to 5 = *almost always/always*.

#### Sociosexual Orientation Inventory-Revised

The Sociosexual Orientation Inventory Revised (SOI-R; Penke and Asendorpf, 2008) has nine items that assess sociosexual orientation based on three dimensions: Behavior (e.g., “With how many different partners have you had sexual intercourse without having an interest in a long-term committed relationship with this person?”; α = 0.92), Attitudes (e.g., “Sex without love is OK”; α = 80), and Desire (e.g., “How often do you have fantasies about having sex with someone with whom you do not have a committed romantic relationship?”; α = 84). These items are rated on a nine-point scale, ranging from 1 = *0* to 9 = *20 or more* in the Behavior factor; from 1 = *strongly disagree* to 9 = *strongly agree* in the Attitudes factor; and from 1 = *never* to 9 = *at least once a day* in the Desire factor. We used the Spanish validation (Barrada et al., 2018). Information about the psychometric properties of the SOI-R in previous samples can be found in Penke (2020).

#### Short version of the Sexuality Scale

Short version (Wiederman and Allgeier, 1993) of the Sexuality Scale (SSS; Snell and Papini, 1989) has 15 items that assess perceptions of one's sexuality through three components: Self-Esteem as a Sexual Partner (e.g., “I am a good sexual partner”; α = 0.89), Dissatisfaction with Sexual Life (e.g., “I'm depressed about the sexual aspects of my life”; α = 0.92), and Sexual Preoccupation (e.g., “I'm constantly thinking about having sex”; α = 90). The items are rated on a five-point Likert-type scale ranging from −2 = *strongly disagree* to +2 = *strongly agree*. Information about the psychometric properties of the Sexuality Scale in previous samples can be found in Snell and Skakoon-Sparling (2020).

#### Control Question

Embedded in the SSS as its sixteenth item to check whether the participants paid enough attention to the item wordings, we introduced an item asking the participants to respond to it with *slightly disagree*.

#### Translation and Adaptation of the SMS and SSS

The English version of the SMS and the SSS were translated into Spanish by two experts in sexuality research using a forward translation procedure. Both the translated and the original versions were given to a bilingual expert in translating psychological and sexological manuscripts to ensure the correspondence between the two versions. Then, the Spanish translation was analyzed by two experts in psychological assessment and sexuality research to identify and suggest changes to items that were not clear or understandable. No changes were made at this phase of the study. Finally, the resulting version was given to two individuals with characteristics similar to the final sample. They received the same task as the experts in psychological assessment and sexuality research. No changes were made at this phase either. The Spanish version of both the SMS and SSS are included as Supplementary Materials.

### Data Analysis

First, we tested the internal structure of the different questionnaires. We did so with confirmatory factor analysis (CFA) and exploratory structural equation modeling (ESEM) approaches (Asparouhov and Muthén, 2009). ESEM is a technique that, unlike CFA, permits all items to load on all factors, and, unlike exploratory factor analysis, permits the correlation between item uniquenesses. Although common CFA models are more parsimonious than ESEM (as no cross-loadings are specified), CFA results can distort inter-factor correlations and loading sizes (Asparouhov and Muthén, 2009; Garrido et al., 2020). ESEM models are thus preferred over CFA ones when they yield better fits, when substantial cross-loadings exist, or when inter-factor correlations differ among solutions (Barrada et al., 2019a).

Second, we modeled the responses to the items from all the questionnaires simultaneously with all the manifest variables (gender, age, being in a relationship, sexual orientation, frequency of masturbation, and age of sexual initiation). In the measurement model, all the factors and manifest variables were allowed to correlate with each other. The latent factors included in the model were those finally retained (five instead of six; see Results section) from SMS, three from SSS, and three from SOI-R. For all the questionnaires, the ESEM model was preferred over the CFA model (see Results section), and all the inter-item correlations of the different questionnaires were modeled using ESEM. In this model, each questionnaire defined a separate set of factors, that is, although cross-loadings in the ESEM models were allowed within each questionnaire, cross-loading was not allowed between different questionnaires (i.e., Item 1 of the SMS is allowed to load on all the factors of the SMS ESEM set, but not on SOI-R or SSS factors). In the structural model, frequency of masturbation, age of sexual initiation, SOI-R factors, and SSS factors were predicted by gender, age, being in a relationship, sexual orientation, and the five sexual motive factors.

The global fit of all the derived models was assessed with the common cut-off values for the fit indices (Hu and Bentler, 1999): CFI and TLI with values >0.95 and RMSEA <0.06 are indicative of a satisfactory fit. It should be noted that these cut-offs were developed for CFAs with continuous responses, so these values should be interpreted with caution (Xia and Yang, 2019). Additionally, these cut-off values should be considered as rough guidelines and not interpreted as “golden rules” (Marsh et al., 2004). Local fit was evaluated using modification indices (MI). New parameters were incorporated into the models one at a time (starting from the largest MI). To avoid problems of capitalization on chance with these re-specifications of the models, we only added new parameters that could be clearly interpreted (Silvia and MacCallum, 1988).

For all the models, the diagonally weighted least squares estimator (WLSMV in M*Plus*) was used. By using this estimator, we were able to maintain the categorical nature of the responses (Finney and DiStefano, 2013). For all the factor models, we interpreted the standardized solution (STDYX solution in M*Plus*). The correlations between dichotomous variables (gender, sexual orientation, being in a relationship) and the different sexual motives were transformed to Cohen's *d* (McGrath and Meyer, 2006) to facilitate their interpretation.

All the latent models were estimated with M*Plus* 7.4 (Muthén and Muthén, 2015). The rest of the analyses were performed with R 3.6.1 (R Core Team, 2020). We used the packages *psych* version 1.8.12 (Revelle, 2018) and *MplusAutomation* version 0.7 (Hallquist and Wiley, 2018). No missing data were present in our database. The open database and code files for these analyses are available at the Open Science Framework repository (https://osf.io/twbqd/).

## Results

### Internal Structure of the Different Instruments

#### Internal Structure of the SMS

We started by testing a CFA and an ESEM model for the SMS items. Results of model fit for these and the next models can be found in Table 1. It can be seen that (a) the ESEM model clearly outperformed the CFA model in terms of fit (ΔCFI = 0.046, ΔTLI = 0.047, ΔRMSEA = −0.034), and (b) this model provided overall satisfactory model fit (CFI = 0.992, TLI = 986, RMSEA = 0.031). Given these results, we proceeded with the ESEM approach for the SMS. The higher modification index indicated the appropriateness of correlating item uniquenesses from Item 3 and Item 15 (MI = 67.2). The two items only differed by a single word in their drafting (“Just for the thrill [excitement] of it”). After testing a model with this additional estimated parameter, the next highest modification index (MI = 21.5) led us to correlate item uniquenesses from Item 2 (“To prove to yourself that your partner thinks you're attractive”) and Item 20 (“To reassure yourself that you are sexually desirable”). No additional correlation among uniquenesses was added, as the next one implied correlating items tapping different factors (MI = 18.1, Item 9 and Item 10).

**Table 1:** Goodness -of- fit indices for the different models.

**Model**	**χ^**2**^**	***df***	***p***	**CFI**	**TLI**	**RMSEA**
M01. SMS CFA	1606.2	362	<0.001	0.946	0.939	0.065
M02. SMS ESEM	435.5	247	<0.001	0.992	0.986	0.031
M03. SMS ESEM CU 03 & 15	375.6	246	<0.001	0.994	0.991	0.026
M04. SMS ESEM CU 03 & 15, 02 & 20	349.1	245	<0.001	0.995	0.992	0.023
M05. Short SMS ESEM CU 03 & 15	270.7	165	<0.001	0.995	0.991	0.028
M06. SOI-R CFA	206.2	24	<0.001	0.990	0.986	0.097
M07. SOI-R ESEM	13.1	12	0.362	1.000	1.000	0.011
M08. SSS CFA	966.9	87	<0.001	0.963	0.955	0.112
M09. SSS ESEM	559.5	63	<0.001	0.979	0.965	0.099
M10. SSS ESEM CU 02 & 15	260.2	62	<0.001	0.992	0.986	0.063
M11. Short SMS + SOI-R + SSS + SOCIO	1583.9	1133	<0.001	0.991	0.989	0.022

*df, degrees of freedom; CFI, comparative fit index; TLI, Tucker-Lewis index; RMSEA, root mean square error of approximation; CFA, confirmatory factor analysis; ESEM, exploratory structural equation modeling; CU, correlated uniquenesses; SMS, Sexual Motivations Scale; SOI-R, Sociosexual Orientation Inventory-Revised; SSS, Short version of the Sexuality Scale; SOCIO, sociodemographic variables (gender, age, being in a relationship, sexual orientation, frequency of masturbation, age of sexual initiation)*.

Although this model offered an excellent model fit (CFI = 0.995, TLI = 0.992, RMSEA = 0.023), it presented important problems in terms of interpretation. Whereas, five out of six intended factors could be clearly identified, the Self-Affirmation factor showed two main problems. First, of the five items intended to measure this motivation, four items presented item loadings on the target factor lower than 0.36 (loadings shown in Table 2). Second, four relevant cross-loadings (range = [0.31, 0.66]) were found. Given that no item was properly measuring this motive, we decided to discard all five items measuring this dimension. We then tested the factor structure of a shortened SMS version.

**Table 2:** Item loadings and interfactor correlations for the Sexual Motivations Scale.

	**Initial SMS—Six factors**						**Short SMS—Five factors**				
**How often do you have sex…**	**PRE**	**INT**	**APP**	**COP**	**ENH**	**AFF**	**PRE**	**INT**	**APP**	**COP**	**ENH**
01. Because others will kid you if you don't?	**0.89**	0.10	0.03	−0.09	−0.09	−0.15	**0.86**	0.08	0.00	−0.17	−0.12
19. Because you worry that people will talk about you if you don't have sex?	**0.94**	−0.02	0.00	0.16	0.03	−0.06	**0.93**	−0.01	−0.01	0.08	0.02
07. Because people will think less of you if you don't?	**0.81**	0.02	0.04	−0.05	−0.10	0.25	**0.88**	0.04	0.07	−0.03	−0.05
13. So that others won't put you down for not having sex?	**0.81**	−0.09	0.11	0.08	−0.03	0.05	**0.85**	−0.08	0.11	0.03	−0.02
25. Just because all your friends are having sex?	**0.77**	−0.12	−0.06	0.15	0.02	0.21	**0.85**	−0.08	−0.03	0.13	0.07
22. To feel emotionally close to your partner?	−0.01	**0.94**	−0.04	0.03	−0.03	0.03	0.01	**0.95**	−0.03	0.02	−0.03
28. To become closer to your partner?	−0.12	**0.90**	−0.01	0.01	−0.03	0.13	−0.05	**0.92**	−0.01	0.04	0.00
04. To connect emotionally with your partner?	0.07	**0.86**	−0.05	−0.02	0.01	−0.06	0.02	**0.85**	−0.04	−0.05	0.00
10. To express love for your partner?	−0.04	**0.77**	0.05	−0.02	0.02	−0.09	−0.07	**0.76**	0.04	−0.05	0.00
16. To become more intimate with your partner?	0.03	**0.74**	0.07	0.06	0.09	0.03	0.05	**0.75**	0.07	0.05	0.10
12. Out of fear that your partner won't love you anymore if you don't?	−0.06	0.02	**0.94**	0.04	−0.02	0.03	−0.03	0.03	**0.94**	0.06	−0.01
06. Because you're worried that your partner won't want to be with you if you don't?	−0.04	0.01	**0.90**	−0.01	−0.03	0.09	−0.01	0.02	**0.90**	0.02	−0.01
24. Because you're afraid that your partner will leave you if you don't?	0.14	0.01	**0.88**	−0.02	0.04	−0.04	0.15	0.01	**0.87**	−0.04	0.04
18. Because you don't want your partner to be angry with you?	0.04	−0.07	**0.82**	0.03	−0.03	−0.07	0.04	−0.07	**0.81**	0.00	−0.03
23. Because it helps you feel better when you're feeling low?	−0.12	0.00	0.02	**0.93**	−0.05	−0.04	−0.10	0.01	0.03	**0.91**	−0.04
17. To cope with upset feelings?	0.06	0.07	0.04	**0.78**	−0.01	−0.17	0.03	0.07	0.03	**0.73**	−0.03
29. To help you deal with disappointment in your life?	0.01	−0.09	0.10	**0.77**	−0.06	0.04	0.03	−0.08	0.11	**0.77**	−0.04
11. To cheer yourself up?	−0.05	0.08	−0.08	**0.76**	0.05	0.00	−0.02	0.10	−0.07	**0.73**	0.07
05. Because it helps you feel better when you're lonely?	0.09	−0.05	−0.07	**0.73**	0.04	0.08	0.14	−0.02	−0.05	**0.72**	0.07
21. Because you feel “horny?”	−0.02	−0.06	0.08	−0.06	**0.88**	−0.01	−0.03	−0.05	0.07	−0.06	**0.88**
27. To satisfy your sexual needs?	−0.08	−0.06	−0.07	0.02	**0.74**	0.07	−0.05	−0.04	−0.07	0.04	**0.76**
09. Because it feels good?	−0.17	0.05	−0.06	0.03	**0.70**	−0.14	−0.21	0.05	−0.08	0.01	**0.67**
03. Just for the excitement of it?	0.23	0.04	−0.01	−0.04	**0.70**	0.02	0.20	0.04	−0.01	−0.03	**0.68**
15. Just for the thrill of it?	0.21	0.04	0.07	0.18	**0.48**	0.03	0.19	0.05	0.08	0.18	**0.48**
26. Because it makes you feel more self—confident?	−0.06	0.04	0.01	**0.48**	−0.01	**0.57**	—	—	—	—	—
20. To reassure yourself that you are sexually desirable?	0.23	0.07	0.21	0.24	0.11	**0.36**	—	—	—	—	—
08. Because it makes you feel like you're a more interesting person?	0.13	−0.03	**0.31**	0.23	−0.01	**0.34**	—	—	—	—	—
14. To help you feel better about yourself?	0.03	0.00	−0.01	**0.66**	0.04	**0.31**	—	—	—	—	—
02. To prove to yourself that your partner thinks you're attractive?	0.15	0.26	**0.37**	−0.02	0.02	0.30	—	—	—	—	—
**Interfactor correlations**	PRE	INT	APP	COP	ENH	AFF	PRE	INT	APP	COP	ENH
PRE											
INT	−0.11						−0.11				
APP	0.60	0.07					0.60	0.06			
COP	0.29	0.13	0.26				0.32	0.12	0.22		
ENH	−0.22	0.18	−0.21	0.21			−0.19	0.17	−0.21	0.20	
AFF	0.27	0.12	0.29	0.42	0.19		—	—	—	—	—

*INT, Intimacy Motives; ENH, Enhancement Motives; AFF, Self-Affirmation Motives; COP, Coping Motives; PRE, Peer Pressure Motives; APP, Partner Approval Motives*.

*Shaded cells indicate the factor where the item theoretically belongs. Bold loadings indicate loadings over |0.30|. Underlined values indicate secondary loadings over |0.30|. Items are ordered by factor loading in the Short SMS solution*.

The ESEM model for this abbreviated version also showed a very good fit (CFI = 0.995, TLI = 0.991, RMSEA = 0.028). In this model, target loadings were, on average, very high (M_loading_ = 0.81, range = [0.48, 0.95]); non-target loadings were small (M_|loading|_ = 0.05, range = [0.00, 0.21]). In this model, the correlation between uniquenesses of Item 3 and Item 15 was equal to 0.52. Excluding Peer Pressure and Partner Approval motives (r = 0.60), the other pair of motives presented a small overlap (rs in the range [−0.21, 0.32]).

#### Internal Structure of the SOI-R and SSS

As the validation of these instruments is not the focus of the present study, we will only briefly comment that, following Barrada et al. (2018), an ESEM model for the SOI-R responses showed a notable improvement in fit over the CFA model (ΔCFI = 0.010, ΔTLI = 0.014, ΔRMSEA = −0.086). For the SSS, we also found evidence for preferring an ESEM model (ΔCFI = 0.016, ΔTLI = 0.010, ΔRMSEA = −0.013). A very large modification index was detected (MI = 330.9), indicating that the correlation between uniquenesses of Item 2 (“I feel good about my sexuality”) and Item 15 (“I feel pleased with my sex life”) should be freely estimated. When estimated, this correlation was equal to 0.55. Thus, for all the considered scales, an ESEM approach was selected. As the SSS was translated into Spanish for this research, item loadings for this scale are provided in the Appendix in Supplementary Material.

### Relations Between Variables

#### Measurement Model

Model fit for this model was very good (CFI = 0.991, TLI = 0.989, RMSEA = 0.022). The association between variables can be seen in Table 3. Describing only the effect sizes in terms of Cohen's d equal to or larger than |0.20| (all those effects were statistically significant, p < 0.05; this cut-point is arbitrary and intended only to simplify results description), men presented a higher mean value on Peer Pressure, d = 0.54, and Coping motives, d = 0.33; participants in a relationship presented higher a mean in Intimacy, d = 0.69, and a lower one in Peer Pressure, d = −0.57, and Coping motives, d = −0.61; non-heterosexual participants presented higher means in Peer Pressure, d = 0.29, and Coping motives, d = 0.20.

**Table 3:** Associations of the different sexual motives.

	**PRE**	**APP**	**COP**	**INT**	**ENH**
**Cohen's** **ds**					
Men	**0.54**	0.01	**0.33**	0.04	0.02
Relation	**−0.57**	−0.03	**−0.61**	**0.69**	−0.04
Hetero	**−0.29**	−0.09	**−0.20**	0.15	−0.17
**Pearson's correlations**					
Age	−0.05	0.06	**−0.09**	**0.12**	−0.02
Age of sexual initiation	**0.15**	0.04	−0.02	0.00	**−0.12**
Frequency of masturbation	**0.12**	−0.06	**0.09**	**−0.12**	**0.09**
SOI–R behavior	0.05	−0.02	**0.24**	**−0.17**	**0.17**
SOI–R attitudes	0.00	**−0.19**	**0.14**	**−0.27**	**0.34**
SOI–R desire	**0.28**	**0.10**	**0.40**	**−0.21**	**0.15**
SSS Self–Esteem as sexual partner	**−0.39**	**−0.35**	−0.04	**0.19**	**0.31**
SSS Dissatisfaction with sexual life	**0.56**	**0.36**	**0.27**	**−0.21**	**−0.15**
SSS Preoccupation with sex	**0.15**	0.02	**0.34**	−0.04	**0.23**

*Men is a dummy variable where 0 = woman and 1 = man. Relation is a dummy variable where 0 = not in a romantic relationship and 1 = in a romantic relationship. Hetero is a dummy variable where 0 = sexual minority and 1 = heterosexual*.

*PRE, Peer Pressure Motives; APP, Partner Approval Motives; COP, Coping Motives; INT, Intimacy Motives; ENH, Enhancement Motives; SOI-R, Sociosexual Orientation Inventory-Revised; SSS, Short version of the Sexuality Scale. Bold values correspond to statistically significant associations, p < 0.05*.

Using as cut-off point |*r*| = 0.20, neither age, age at sexual initiation, nor frequency of masturbation presented relevant correlations with sexual motives. Peer Pressure motives were positively related to sociosexual Desire (*r* = 0.28) and to Dissatisfaction with Sexual Life (*r* = 0.56); and negatively to Self-Esteem as Sexual Partner (*r* = −0.39). Partner Approval motives were positively related to Dissatisfaction with Sexual Life (*r* = 0.36), and negatively to Self-Esteem as Sexual Partner (*r* = −0.35). Coping motives showed positive correlations with sociosexuality (*r* = 0.24 for Behavior and *r* = 0.40 for Desire), with Dissatisfaction with Sexual Life (*r* = 0.27), and with Preoccupation with Sex (*r* = 0.34). Intimacy motives presented negative relations with sociosexuality dimensions (*r* = −0.27 for Attitudes and *r* = −0.21 for Desire), and with Dissatisfaction with Sexual Life (*r* = −0.21). Enhancement motives presented positive relations with sociosexuality (*r* = 0.34 for Attitudes), with Self-Esteem as Sexual Partner (*r* = 0.31), and with Preoccupation with Sex (*r* = 0.23).

#### Structural Model

As the structural model was a saturated model (no path between variables omitted), model fit was equal to fit in the measurement model. For simplicity, we will restrict the description of these results to standardized coefficients larger than |0.15| and only for sexual motive dimensions. Complete results are shown in Table 4.

**Table 4:** Structural model of the relationships between the five sexual motives and sociodemographic data with sexual behavior, sociosexuality, and psychosexual measures.

	**Frequency of Masturbation**			**Age of at Sexual Initiation**			**SOI–R Behavior**			**SOI–R Attitudes**		
*R*^2^	0.206			0.092			0.287			0.290		
**Coefficients**	**beta**	***z***	***p***	**beta**	***z***	***p***	**beta**	***z***	***p***	**beta**	***z***	***p***
Men	**0.37**	**19.01**	**<0.001**	0.06	1.53	0.126	0.04	0.91	0.361	0.05	1.38	0.169
Age	−0.02	−0.81	0.418	**0.22**	**7.47**	**<0.001**	**0.35**	**10.86**	**<0.001**	0.07	1.92	0.055
Relation	−0.08	−1.73	0.084	−0.02	−0.58	0.563	**−0.22**	**−5.75**	**<0.001**	**−0.10**	**−2.36**	**0.018**
Hetero	**−0.11**	**−5.17**	**<0.001**	0.00	−0.08	0.934	**−0.09**	**−2.56**	**0.011**	**−0.11**	**−3.01**	**0.003**
PRE	0.06	0.89	0.372	**0.19**	**2.29**	**0.022**	−0.04	−0.54	0.588	0.06	0.62	0.538
APP	−0.08	−1.32	0.187	−0.11	−1.54	0.124	−0.04	−0.58	0.560	**−0.17**	**−2.12**	**0.034**
COP	0.00	−0.05	0.963	−0.03	−0.56	0.577	**0.21**	**4.94**	**<0.001**	0.09	1.68	0.092
INT	**−0.09**	**−2.41**	**0.016**	0.03	0.72	0.470	**−0.19**	**−4.82**	**<0.001**	**−0.31**	**−7.75**	**<0.001**
ENH	**0.09**	**2.51**	**0.012**	**−0.10**	**−2.28**	**0.023**	**0.14**	**3.42**	**0.001**	**0.34**	**7.61**	**<0.001**
	**SOI–R desire**			**SSS Self–Esteem**			**SSS dissatisfaction**			**SSS preoccupation**		
*R*^2^	0.417			0.270			0.395			0.184		
**Coefficients**	**beta**	***z***	***p***	**beta**	***z***	***p***	**beta**	***z***	***p***	**beta**	***z***	***p***
Men	**0.24**	**7.08**	**<0.001**	0.03	0.70	0.485	0.00	−0.06	0.956	**0.12**	**3.26**	**0.001**
Age	−0.04	−1.15	0.250	0.07	1.82	0.069	0.02	0.49	0.624	−0.05	−1.36	0.175
Relation	**−0.32**	**−9.52**	**<0.001**	**0.18**	**4.42**	**<0.001**	**−0.20**	**−4.68**	**<0.001**	0.04	0.95	0.343
Hetero	**−0.09**	**−2.80**	**0.005**	0.00	0.04	0.968	−0.03	−0.82	0.412	0.02	0.54	0.593
PRE	0.04	0.49	0.622	**−0.19**	**−2.57**	**0.010**	**0.41**	**5.38**	**<0.001**	0.12	1.48	0.139
APP	0.05	0.84	0.402	**−0.21**	**−3.33**	**0.001**	0.07	1.09	0.276	−0.06	−0.82	0.410
COP	**0.23**	**5.72**	**<0.001**	0.07	1.40	0.161	**0.11**	**2.11**	**0.035**	**0.28**	**5.37**	**<0.001**
INT	**−0.15**	**−4.49**	**<0.001**	0.07	1.63	0.102	**−0.10**	**−2.43**	**0.015**	**−0.11**	**−2.54**	**0.011**
ENH	**0.13**	**3.52**	**<0.001**	**0.21**	**4.96**	**<0.001**	−0.07	−1.46	0.144	**0.21**	**4.16**	**<0.001**

*Men is a dummy variable where 0 = woman and 1 = man. Relation is a dummy variable where 0 = not in a romantic relationship and 1 = in a romantic relationship. Hetero is a dummy variable where 0 = sexual minority and 1 = heterosexual. PRE, Peer- Pressure Motives; APP, Partner- Approval Motives; COP, Coping Motives; INT, Intimacy Motives; ENH, Enhancement Motives; SOI-R, Sociosexual Orientation Inventory-Revised; SSS, Short version of the Sexuality Scale; Self-Esteem, Self-Esteem as Sexual Partner; Dissatisfaction, Dissatisfaction with Sexual Life; Preoccupation, Preoccupation with Sex. Bold values indicate statistically significant coefficients p < 0.05*.

No sexual motive was relevantly related to frequency of masturbation. Higher scores in Peer Pressure were related to later sexual initiation, β = 0.19, lower Self-Esteem as Sexual Partner, β = −0.19, and higher Dissatisfaction with Sexual Life, β = 0.41. Partner Approval motives were negatively related to sociosexual Attitudes, β = −0.17, and Self-Esteem as Sexual Partner, β = −0.21. Intimacy motives were negatively related to all three sociosexual dimensions, βs in the range [−0.31, −0.15]. Coping motives were positively related to sociosexual Behavior, β = 0.21, to Desire, β = 0.23, and to Preoccupation with Sexual Life, β = 0.28. Enhancement motives were positively related to more positive sociosexual Attitudes, β = 0.34, higher Self–Esteem as Sexual Partner, β = 0.21, and Preoccupation with Sexual Life, β = 0.21. The percentage of explained variance ranged from 9.2% for age at sexual initiation to 41.7% for sociosexual Desire.

## Discussion and Conclusions

Knowledge of the diverse sexual motivations can have profound implications for our comprehension of the causes, correlates, and consequences of sexual behavior (Cooper et al., 2011). Because of this relevance, and trying to overcome some limitations in the literature, this study had a dual objective. On the one hand, to validate the SMS (Cooper et al., 1998) in Spanish, offering a valid and reliable instrument that facilitates future research in Spanish-speaking contexts and comparability of the results while using a better psychometric approach in comparison with previous studies. On the other hand, to determine Spanish university students' sexual motivations and their relationship with different sociodemographic and psychosexual variables and with sexual behavior.

Based on the results obtained, different conclusions can be drawn, and topics can be raised for discussion, both from a psychometric and theoretical point of view. As for the instrument used, the Spanish validation of the SMS was performed, which implies the first validation of this scale in a language other than English and outside the United States. A more refined analysis has been carried out, illustrating the use of ESEM and how it can lead to clearly different decisions than CFA. In other words, the use of CFA can lead to erroneous conclusions, as relevant cross-loadings may remain undetected by global model fit indices, so the more flexible ESEM technique should be preferred. In fact, the CFA model fit reported by Jardin et al. (2017) was almost equivalent to the fit of our tested CFA model (Jardin et al./present results: CFI = 0.946/0.946, RMSEA = 0.075/0.065), but we included an additional analysis that indicated the presence of relevant cross-loadings, greatly improved model fit, and led to theoretical insights. If we had only performed CFA, the problems with the Self-Affirmation dimensions could have been missed.

Our findings seem highly consistent with findings reported in North America. The structure is highly similar and is in line with the theoretical structure expected by Cooper et al. (1998). Five of the six factors replicated perfectly, and the patterns of correlates also seem mostly similar. However, as a result of our analysis, a new version of the SMS is proposed, in which we suggest discarding the subscale of Self-Affirmation, because of its items' cross-loading with other factors, specifically those of Coping and Partner approval. Apparently, although we can distinguish conceptually between doing something so as not to feel bad and to feel good, participants consider all this as a single dimension. Recently, Barrada et al. (2019a) found a similar result in a gambling motives scale where both kinds of motives collapsed into a general affect-regulation dimension.

With the available data, it is not possible to offer a clear explanation for the differences between the factor analysis by Cooper et al. (1998) or Jardin et al. (2017) and our analysis. As Hirschfeld et al. (2014) and Garrido et al. (2020) have noted, in factor analysis and in some conditions, thousands of participants would be required to obtain stable loading patterns, so a possible explanation for the differences could be sample error. Alternative explanations may be differences due to the language or cultural background of the participants. From our point of view, part of the differences may be due to the different analytical approaches, with our selected technique (ESEM; not available more than 20 years ago) better suited to recover the internal structure of the scores. Also some tentative explanations can be offered by inspection of the items' wording. Some items of the Self-Affirmation dimension addressed, at least partially, partner approval (e.g., “To prove to yourself that your partner thinks you're attractive?”), so a relevant cross-loading is not surprising. In this line, motives for not feeling bad (Coping) or for feeling better (Self-Affirmation), although conceptually separable, have been found to collapse into a single dimension in other questionnaires (Barrada et al., 2019a). In any case, these differences indicate the appropriateness of replicating studies of the psychometric properties of commonly used instruments.

Thus, a scale of 24 items, with five factors, is proposed. The subscales of appetitive self-focused motives (Enhancement) and social motives are maintained, and both appetitive (Intimacy) and aversive (Peer Pressure and Partner Approval) motives remain, but the subscale of aversive self-focused motives includes only Coping, removing Self-Affirmation. With this proposed new structure, the SMS is considered a valid and reliable tool to measure sexual motivations also in Spanish.

As for Spanish university students' reasons for having sex, this study makes several contributions. First, the reasons' association both with sociodemographic variables (i.e., sex, age, sexual orientation, relational status) and with behavioral variables (i.e., frequency of masturbation, age at initiation, sociosexual behavior), disposition (i.e., sociosexual attitudes and desire), and well-being (i.e., self-esteem as a sexual partner, satisfaction with sex life, preoccupation with sex). Secondly, the relevance of certain motivations, such as those of coping, peer pressure, and enhancement concerning the variables evaluated, has been revealed. Interestingly, the positive motives presented opposite correlations with sociosexual orientation, negative for Intimacy and positive for Enhancement. Both positive dimensions were positively associated with the measures that more clearly tapped sexual well-being, Self-Esteem as Sexual Partner and (Dis)satisfaction with Sexual Life. This provides further evidence that these two dimensions—both appetitive motivations—can and should be differentiated. Overall, the aversive dimensions shared negative associations with sexual well-being.

It is important to underline peer-pressure motives, which were not taken into account in different developments based on the SMS (Patrick et al., 2011; Kenney et al., 2014; Snapp et al., 2014) because they were considered to be less relevant and to have a lower association with other constructs than the subscales of coping, intimacy, and enhancement. In other age groups with older participants, peer-pressure motives may be less relevant, but the results obtained in this study show their importance in the life of emerging adults and the need to take them into account because of their relationship with the other variables evaluated (see Tables 3, 4).

As for sociodemographic variables, men were found to score significantly higher than women on aversive sexual motivations, both individual (coping) and social (peer pressure). The same is true among non-heterosexuals compared to heterosexuals. This may indicate that women and heterosexual people make more adaptive use of sex and that men and non-heterosexual people may be more dissatisfied with their sex life, although this was not observed in the present study. This result contradicts those found in previous research, especially concerning the differences between men and women (Leigh, 1989; Cooper et al., 1998; Ott et al., 2006; Meston and Buss, 2007; Armstrong and Reissing, 2015) and poses the need to continue investigating along these lines to determine whether this was a punctual result or there is a change of tendency, and women are making a healthier use of sexual relations.

As in previous studies, it was found that, as the age of young people increases, intimacy motivations, and the interest in establishing committed relationships gain importance, while other motivations become less relevant, such as those of coping (Cooper et al., 1998; Ott et al., 2006). The same occurs with peer-pressure motives, more habitual among adolescents and emerging adults, but whose relevance decreases as age increases (Patrick et al., 2011; Kenney et al., 2014; Snapp et al., 2014). Here, it should be noted that, as classical literature affirms (Leigh, 1989; Hill and Preston, 1996), procreation is a very powerful reason for having sex, but it usually appears at older ages than those of the participants in this study.

High and expected associations (Cooper et al., 1998; Armstrong and Reissing, 2015; Gravel et al., 2016; Jardin et al., 2017) between relational status and sexual motivations were found, such that participants who had no partner presented higher scores than those who had a partner for aversive motivations, such as those of coping and peer pressure, and lower scores in appetitive motivations, such as those of intimacy. It is considered that having a partner acts as a protective factor and has positive consequences for well-being (Debrot et al., 2017), as can be seen in this study, with higher scores in self-esteem as a sexual partner and satisfaction with sex life in people who had a partner.

As for sexual behavior, frequency of masturbation has been poorly assessed concerning sexual motives. The results of our study conclude that it is associated with almost all types of evaluated motivations, that is, masturbation is a resource that relates both to the adaptive elements of sex (more enhancement, less intimacy) and to the aversive elements (more coping and peer pressure). As expected, the age at initiation was directly related to peer-pressure motives and indirectly to enhancement motives, as there is a significant proportion of young people who initiate sex because their peers do it and not because they feel safe or enjoy it (Cooper et al., 1998; Sieving et al., 2006; Grossbard et al., 2007; Patrick et al., 2007).

A unique contribution of this study in relation to others carried out previously (Cooper et al., 1998; Meston and Buss, 2007), with the exception of that of Meston et al. (2020), is the consideration of sociosexuality as a three-dimensional concept, made up of behavior, attitudes, and desire (Penke and Asendorpf, 2008; Barrada et al., 2018). Similar relationships were found in the three dimensions of the construct (direct relationships with coping and enhancement motivations, reverse relationships with intimacy motivations), and with more powerful associations in the case of sociosexual desire. These associations, both with aversive and appetitive motivations, allow us to conclude that casual sex can be used both to obtain pleasure and to conceal negative emotions (Correa et al., 2017).

The last of the contributions to be highlighted is the relationship found between motivations for sex and psychosexual well-being. Similar patterns were found in the evaluated variables, such that people who had sex for positive reasons, both individual (enhancement) and social (intimacy), showed more sexual self-esteem and less dissatisfaction with sex life. Individuals who had sex for aversive reasons showed the opposite results. As for preoccupation with sex, being a neutral construct, it does not provide very conclusive results. These novel results justify *per se* the need and relevance of knowing people's motives for having sex. Also, they have important implications for the work of clinicians and professionals of prevention and promotion of sexual health and can serve as the basis for future research. If, as found, there is a relationship between reasons for having sex and psychological well-being/ill-being, the work of these professionals could focus on promoting positive motivations and reducing negative ones to achieve an individual's better sexual well-being.

The study has a number of limitations, mainly related to the representativeness of the sample and the generalization of the results. Among the participants, the sample was mostly female, late adolescents, and emerging adults, aged 18 and 26, and from a single Spanish university, making the results difficult to generalize to all university students and still less to other groups of the same or different ages. Concerning our exclusion criteria, data from the SMS were collected only from participants who had had any sexual intercourse with penetration in their lifetime. Although this is a restrictive conception of sexuality, by doing so, we intended to establish a clearer operationalization of what could be considered as “sexually experienced.” Future studies should consider including participants with non-penetrative sexual experiences such as masturbation and oral sex. Also, we only included participants labeling themselves as a woman or a man. Studying other gender identities (Hyde et al., 2019) should be considered in further research. Regarding sexual orientation, to facilitate the analysis, it was decided to group the participants into heterosexual and non-heterosexual, which led to the loss of relevant information about the behavior patterns and motivations of minority sexual members. Also, our study shares with other studies based on self-selected samples and self-reported measures the fact that the results may be limited by response and recall bias. Finally, a limitation that our study shares with most of the existing literature on the subject is that it is a cross-sectional study. It would be interesting to carry out longitudinal studies, which would allow analyzing whether sexual motivations change across youth, in what direction, whether they influence relational status, and how this relates to sexual behavior and psychosexual well-being. It would also be interesting to examine the reasons why some people choose not to have sex, thereby expanding the range of participants.

Despite these limitations, the study is considered to make important contributions, in some cases, to aspects about which the literature is scarce. This is the first validation of the SMS outside of North America, providing a valid and reliable instrument to assess sexual motives in Spanish-speaking contexts, which will facilitate further research. As a result of this process, significant changes and improvements for the SMS have been proposed. The techniques used through the analysis have revealed some problems with one of the factors. It is important to note that, through a CFA, these problems would not have been detected (the CFA model presented a fit that, in many studies, would be considered acceptable; Jackson et al., 2009). Also, we relied on latent variables (factor analyses, structural equation modeling) rather than manifest ones (sum of items). By doing so, we reduced the risk of the results being affected by measurement error (Cole and Preacher, 2014).

Moreover, this is also the first study carried out on the sexual motivations of young Spaniards, analyzing the differences in these motivations based on sex, age, relational status, and sexual orientation. Information has been provided on the associations between sociosexuality, understood as a three-dimensional construct (behavior, attitudes, desire), and motivations for having sex. We have noted possible associations between sexual motivations and psychosexual well-being, understood as self-esteem as a partner, satisfaction with sex life, and preoccupation with sex, finding novel results about the nature of the reasons for having sex (positive/aversive) and higher or lower psychosexual well-being. All these contributions and the implications of the results obtained are relevant both for researchers and for clinicians and professionals of prevention and promotion of sexual health. Knowing the relevance of the motives for sex and their relationship with other variables, especially well-being, is essential to develop this line of research and to include these variables in the treatments and interventions, to achieve healthy sexual relations and higher psychological and psychosexual well-being.

## Data Availability Statement

The open database and code files for these analyses are available at the Open Science Framework repository (https://osf.io/twbqd/).

## Ethics Statement

The studies involving human participants were reviewed and approved by Ethics Review Board for Clinical Research of Aragón, Spain (ref. PI18/058). The participants provided their informed consent to participate in this study.

## Author Contributions

JB, ÁC, EF-d-R, and PR-V contributed conception, design of the study, and wrote the second draft of the manuscript. JB organized the database and performed the statistical analysis. JB and ÁC wrote the first draft of the manuscript. All authors contributed to manuscript revision, read, and approved the submitted version.

## Conflict of Interest

The authors declare that the research was conducted in the absence of any commercial or financial relationships that could be construed as a potential conflict of interest.
