# Supplementary material for: Motives to Have Sex: Measurement and Correlates With Sociodemographic, Sexual Life, and Psychosexual Characteristics
Source: Front Psychol. 2021 Jul 12;12:645493. doi: 10.3389/fpsyg.2021.645493 (PMC8311163; doi:10.3389/fpsyg.2021.645493)
Supplement: Supplementary file 1 [file Table_1.DOCX]

Supplementary Material

**Sexual Motivations Scale – Spanish Version**

Para cada frase, por favor indica qué número describe mejor con qué frecuencia tienes relaciones sexuales para cada una de las siguientes razones. Recuerda: no hay respuestas correctas o incorrectas. Solo queremos saber lo que piensas.

|  |  | Casi nunca / Nunca | Algunas veces | Alrededor de la mitad de las veces | La mayoría de veces | Casi siempre / siempre |
| --- | --- | --- | --- | --- | --- | --- |
|  | **¿Con qué frecuencia tienes relaciones sexuales...** | | | | | |
| (01) | ... porque la gente se reiría de ti si no lo hicieras? |  |  |  |  |  |
| (02) | ... para demostrarte que tu pareja piensa que eres atractivo/a? |  |  |  |  |  |
| (03) | ... solo por la excitación de hacerlo? |  |  |  |  |  |
| (04) | ... para conectarte emocionalmente con tu pareja? |  |  |  |  |  |
| (05) | ... porque te ayuda a sentirte mejor cuando te sientes solo/a? |  |  |  |  |  |
| (06) | ... porque te preocupa que tu pareja no quiera estar contigo si no lo haces? |  |  |  |  |  |
| (07) | ... porque la gente pensará mal de ti si no lo haces? |  |  |  |  |  |
| (08) | ... porque te hace sentir que eres una persona más interesante? |  |  |  |  |  |
| (09) | ... porque el sexo es placentero? |  |  |  |  |  |
| (10) | ... para expresar amor por tu pareja? |  |  |  |  |  |
| (11) | ... para animarte? |  |  |  |  |  |
| (12) | ... por miedo a que tu pareja no te siga queriendo si no lo haces? |  |  |  |  |  |
| (13) | ... para que la gente no se meta contigo por no tener relaciones sexuales? |  |  |  |  |  |
| (14) | ... para ayudarte a sentirte mejor contigo mismo/a? |  |  |  |  |  |
| (15) | ... solo por la emoción de hacerlo? |  |  |  |  |  |
| (16) | ... para conseguir más intimidad con tu pareja? |  |  |  |  |  |
| (17) | ... para manejar sentimientos negativos? |  |  |  |  |  |
| (18) | ... porque no quieres que tu pareja se enfade contigo? |  |  |  |  |  |
| (19) | ... porque te preocupa lo que la gente pueda decir de ti si no lo haces? |  |  |  |  |  |
| (20) | ... para reafirmarte en que eres sexualmente deseable? |  |  |  |  |  |
| (21) | ... porque te sientes cachondo/a? |  |  |  |  |  |
| (22) | ... para sentirte cercano/a emocionalmente a tu pareja? |  |  |  |  |  |
| (23) | ... porque te ayuda a sentirte mejor cuando te sientes mal? |  |  |  |  |  |
| (24) | ... porque te asusta que tu pareja te deje si no lo haces? |  |  |  |  |  |
| (25) | ... solo porque todos tus amigos/as tienen relaciones sexuales? |  |  |  |  |  |
| (26) | ... porque te da más confianza en ti mismo/a? |  |  |  |  |  |
| (27) | ... para satisfacer tus necesidades sexuales? |  |  |  |  |  |
| (28) | ... para sentirte más cercano/a a tu pareja? |  |  |  |  |  |
| (29) | ... para hacer frente a las decepciones de tu vida? |  |  |  |  |  |

**Short Sexuality Scale – Spanish Version**

SESP = Self-Esteem as a Sexual Partner; DSL = Dissatisfaction with Sexual Life; SP = Sexual Preoccupation

|  |  | SESP | DSL | SP |
| --- | --- | --- | --- | --- |
| (01) I am a good sexual partner | Soy una buena pareja sexual | **0.83** | -0.10 | 0.03 |
| (02) I feel good about my sexuality | Me siento bien con mi vida sexual | 0.11 | **-0.80** | 0.11 |
| (03) I think about sex all the time | Pienso en sexo todo el tiempo | 0.03 | -0.05 | **0.87** |
| (04) I would rate my sexual skill quite highly | Considero que mis habilidades sexuales son muy buenas | **0.86** | 0.00 | 0.06 |
| (05) I am depressed about the sexual aspects of my life | Me deprimen los aspectos sexuales de mi vida | -0.01 | **0.89** | 0.01 |
| (06) I tend to be preoccupied with sex | Tiendo a estar obsesionado/a con el sexo | 0.03 | 0.17 | **0.81** |
| (07) I am disappointed about the quality of my sex life | Me entristece mi vida sexual | 0.07 | **0.98** | 0.02 |
| (08) I think about sex more than anything else | Pienso en sexo más que en ninguna otra cosa | -0.03 | 0.07 | **0.90** |
| (09) I am constantly thinking about having sex | Constantemente pienso en practicar sexo | -0.09 | -0.03 | **0.88** |
| (10) I think of myself as a very good sexual partner | Me considero una pareja sexual muy buena | **0.97** | 0.01 | 0.04 |
| (11) I feel down about my sex life | Me decepciona la calidad de mi vida sexual | 0.10 | **0.96** | -0.01 |
| (12) I think about sex a great deal of the time | Pienso en sexo una gran parte del tiempo | -0.07 | 0.02 | **0.90** |
| (13) I would rate myself low as a sexual partner | Me considero una pareja sexual no muy buena | **-0.55** | 0.35 | 0.04 |
| (14) I am confident about myself as a sexual partner | Confío en mí mismo/a como pareja sexual | **0.68** | -0.22 | -0.02 |
| (15) I feel pleased with my sex life | Me siento satisfecho con mi vida sexual | 0.02 | **-0.80** | 0.06 |
| Interfactor correlations |  | SESP | DSL | SP |
|  | SESP |  |  |  |
|  | DSL | –.51 |  |  |
|  | SP | .13 | .25 |  |

| –2 = | disagree | En total desacuerdo |
| --- | --- | --- |
| –1 = | slightly disagree | Medianamente en desacuerdo |
| 0 = | neither agree nor disagree | Ni de acuerdo ni en desacuerdo |
| +1 = | slightly agree | Medianamente de acuerdo |
| +2 = | agree | Totalmente de acuerdo |
